# Inflammatory Bowel Disease-related Spondyloarthritis: The Last Unexplored Territory of Rheumatology

**DOI:** 10.31138/mjr.33.1.126

**Published:** 2022-04-15

**Authors:** Nikoleta Zioga, Dionysios Kogias, Vasiliki Lampropoulou, Nikolaos Kafalis, Charalampos Papagoras

**Affiliations:** First Department of Internal Medicine, University Hospital of Alexandroupolis, Democritus University of Thrace, Alexandroupolis, Greece

**Keywords:** spondyloarthritis, ankylosing spondylitis, enteropathic arthritis, inflammatory bowel disease, Crohn’s disease, ulcerative colitis

## Abstract

The Spondyloarthritides (SpA) are a group of chronic inflammatory d iseases a ffecting th e spine, peripheral joints and entheses, as well as extra-skeletal structures, including the gastrointestinal tract. On the other hand, inflammatory b owel d isease (IBD), e ither Crohn’s d isease o r ulcerative colitis, often affects extra-intestinal sites, including the axial and/or peripheral skeleton. IBD-related arthritis is the type of SpA that occurs in patients affected by IBD, with an incidence up to 50% during the IBD course. Although both manifestations are apparently the result of a common pathogenetic process, physicians often fail to recognize the disease in its entirety: thus, IBD-SpA is managed as two separate diseases, a musculoskeletal and a gastrointestinal one, with a profound impact on patient quality of life. Moreover, the specialty of the treating physician determines the clinical and laboratory tools for disease assessment, which, in turn, guide treatment decisions that may overlook either affected system or even act in the opposite direction. Raising awareness of the intestinal and musculoskeletal manifestations among rheumatologists and gastroenterologists will lead to earlier diagnosis and a multidisciplinary approach, particularly regarding pharmacologic treatments. Given the lack of trial evidence on immunomodulatory drugs in IBD-SpA it is imperative for researchers in both medical disciplines to join efforts, in order to determine referral strategies, appropriate composite measures for disease assessment, treatment algorithms and therapeutic targets.

## INTRODUCTION

It has been over 90 years since the first report linking ulcerative colitis with arthritis,^1^ and over 60 years, it became evident that the musculoskeletal involvement accompanying ulcerative colitis (UC) and Crohn’s disease (CD) has much in common with ankylosing spondylitis (AS).^2,3^ Following the introduction of the unifying concept of spondyloarthritis (SpA),^4^ the inflammatory bowel disease (IBD) has been regarded as a hallmark extraskeletal feature of SpA and has been incorporated in all sets of SpA classification criteria, including the most recent one proposed by the Assessment of Spondyloarthritis International Society (ASAS).^5–7^

The recognition of the link between intestinal inflammation and joint disease has led to the introduction of the term “enteropathic arthritis”. Although medical textbooks generally reserve this term for the musculoskeletal disease accompanying CD and UC, enteropathic arthritis could mean any type of arthritis associated with intestinal disease. Indeed, the term has also been used to denote joint diseases within the SpA spectrum, such as reactive arthritis, or outside of it, such as arthritis in Whipple’s disease, celiac disease or following intestinal bypass surgery.^8^ It is remarkable that in the 10th revision of the International Classification of Diseases (ICD) that is still broadly used, there are 3 codes for enteropathic arthropathy, ie, “in Crohn disease” (M07.4), “in ulcerative colitis” (M07.5) and “other enteropathic arthropathies” (M07.6). To make things more complicated, in the latest 11^th^ ICD revision, the term “enteropathic arthropathies” is completely eliminated and the CD- and UC-related arthritides are coded with the term “Other specified reactive arthropathies” (FA11.Y). Although this appears a minor detail, it actually preserves the century-old belief that IBD-related arthritis is a mere musculoskeletal reflection of the intestinal inflammation and, therefore, the treatment of IBD is sufficient to address joint disease, as well. This and other misconceptions are probably the reason why IBD-related SpA, perhaps the most appropriate term, has been largely ignored by rheumatologic clinical and translational research, in contrast to its skin inflammatory disease counterpart, ie, psoriatic arthritis (PsA).

## PREVALENCE AND TYPES OF MUSCULOSKELETAL INVOLVEMENT

The introduction of the Amor^5^ and European Spondylarthropathy Study Group (ESSG) criteria^6^, in the early 90’s, for the first time, allowed researchers to formally classify IBD patients with musculoskeletal symptoms as suffering from SpA. In 2009, the ASAS criteria introduced some innovations in the SpA concept that apply to IBD-related SpA as well: the clinical manifestations are distinguished in axial and peripheral, while axial SpA is further divided in radiographic and non-radiographic depending on whether sacroiliac damage is evident on plain radiographs.^7,9^ However, most studies on the epidemiology of IBD-related SpA used the older criteria.^10^ Hence, the overall prevalence of SpA manifestations in IBD patients was estimated between 17%–62% and the prevalence of SpA according to the ESSG criteria between 5%–45.7%.^11^ More recently, in a Norwegian cohort of IBD patients (IBSEN cohort), 36% fulfilled the ASAS criteria for SpA, of whom 8% for axial SpA and 28% for peripheral SpA.^12^

### Axial disease

Axial involvement in IBD has been reported under various definitions, such as AS, sacroiliitis and inflammatory back pain. According to a meta-analysis, the pooled prevalence of AS in IBD is 3% (95% confidence interval [CI] 2–4%).^10^ Conversely, in a meta-analysis of extra-articular manifestations of AS, the prevalence of IBD was estimated at 6.8% (95% CI 6.1%–7.7%).^13^ Sacroiliitis in IBD patients is more frequent, with a pooled prevalence of 10% (95% CI 8–12%), which may be either clinical (pooled prevalence 8%), or subclinical (pooled prevalence 11%).^10^ This variation might be due to IBD treatment with tumour necrosis factor-α (TNFα) inhibitors, which improve the spinal symptoms, but may not equally affect radiographic damage. Finally, the most frequent axial manifestation of IBD is inflammatory back pain (IBP), which has been reported in 5.2–42% of patients.^11^ As in the rest of axial SpA, a possible explanation might be that IBD-related SpA begins with inflammatory back pain followed over the years, at least in a proportion of patients, by structural damage allowing it to be diagnosed as radiographic axial SpA (AS). Although in this regard more sensitive imaging modalities would reveal a higher prevalence of sacroiliitis in the IBD population, sacroiliitis has been identified at similar proportions with conventional radiography (12%), computed tomography (CT) (15%) and magnetic resonance imaging (MRI) (10%) (10). Concerning the type of IBD, it appears that axial manifestations occur slightly more often in CD than UC.^10^

### Peripheral disease

Peripheral arthritis, enthesitis and dactylitis are another type of musculoskeletal involvement in IBD-related SpA. According to the above-mentioned meta-analysis the pooled prevalence of peripheral arthritis in IBD is 13% (95% CI 12–15%) with a slight predominance in CD (15%) than UC (12%).^10^ The incidence of peripheral arthritis was also assessed in the IBSEN cohort of IBD patients who were followed over 20 years. The authors reported that 76 out of 441 patients (17.2%) had ever manifested pure peripheral arthritis that could be attributed to IBD. There was a significant predominance of females (67%), while there was no difference in the prevalence of CD-related and UC-related peripheral arthritis. Finally, another 19 patients (4.3%) had at least one instance of enthesitis or dactylitis.^14^ It should be noted, though, that there is a high variability in the rates of enthesitis across studies, ranging from 1–54% with no clear difference between CD and UC.^10,11^ In contrast, dactylitis has been reported in less than 5% of patients with either CD or UC.^10^

Concerning patients’ perceptions of the impact of the disease on their quality of life, in the IBSEN cohort the presence of arthralgia or back pain was independently associated with worse scores across all domains (except for mental health) of the Short Form 36 (SF-36) questionnaire, as well as with higher levels of fatigue and chronic fatigue.^12^ Moreover, in an Italian cohort of IBD-related SpA, the variables that had a significant positive correlation with function, as assessed with the Health Assessment Questionnaire (HAQ), were the Bath Ankylosing Spondylitis Functional Index (BASFI) and the AS Disease Activity Index-C-Reactive Protein (ASDAS-CRP), but not measures of intestinal disease activity, such as the partial Mayo score, the Crohn’s Disease Activity Index (CDAI) or the IBD Questionnaire (IBDQ).^15^ It is, thus, obvious, that musculoskeletal manifestations are frequent in patients with IBD causing considerable pain and impairment in their quality of life, independently to that inflicted by the IBD itself.

## PATHOPHYSIOLOGICAL LINKS BETWEEN GUT AND SKELETAL INFLAMMATION

The current concept of the pathogenesis of SpA holds that the initial immunological challenge occurs at the level of intestinal mucosa (or the skin in the case of PsA). It is assumed that a particular genetic background allows for an aberrant gut immune response against rather common insults, which eventually spreads and persists at musculoskeletal sites.^16^

Indeed, several studies in which axial SpA patients were screened for gut inflammation by ileocolonoscopy and biopsy or endoscopic capsule raised the prevalence of subclinical gut inflammation to more than 50% of patients with axial SpA, almost 10-fold higher than the reported co-existence of clinical IBD and AS.^17–19^ Regarding peripheral arthritis, Orchard et al. divided IBD-related peripheral arthritis into 2 types: type 1, an oligoarthritis involving large joints of the legs, which typically occurs during periods of active gut disease and wanes off within weeks; and type 2, a chronic arthritis which may involve several joints, including small ones of the hands, and lacks a clear association with the activity of the intestinal disease. In 4–14% of patients with either type, the arthritis occurred before IBD diagnosis, but it is uncertain whether it predated the actual onset of IBD.^20^ These observations suggest that in some IBD patients the arthritis may be a remote repercussion of active gut inflammation, much like reactive arthritis. Importantly, though, in a proportion of IBD patients, arthritis seems to be a separate manifestation of the disease, which, once triggered, follows an independent course from the bowel disease.

From a pathophysiological aspect, several genetic loci related to immune function, cytokine signalling and major histocompatibility complex (MHC) proteins have been associated with either SpA and IBD, with plenty of them being common for both diseases.^21^ Moreover, environmental stresses such a smoking and, particularly, intestinal dysbiosis have been implicated in the pathogenesis of both IBD and SpA, while mechanical forces are related solely to SpA.^16,22,23^

A possible mechanism linking genes, microbiota, intestinal and joint disease is exemplified by the human leukocyte antigen B27 (HLA B27)/β2-microglobulin transgenic rat model of human SpA. In this model, immunological changes in the gut, such as production of interleukin 1β (IL-1β) and TNFα occur early, followed by an increased expression of interferon γ (IFNγ) and type 3 immunity cytokines (IL-23, IL-17). Subsequently, dysbiotic changes and intestinal inflammation develop prior to the ultimate onset of clinical arthritis.^24,25^ In healthy humans, carriage of HLA B27 has also been associated with intestinal microbiota alterations,^26^ while intestinal dysbiosis and subclinical gut inflammation is a feature of AS.^27^ Innate mechanisms, such as autophagy, are involved at the intestinal level both in the subclinical gut inflammation of AS, as well as in active UC.^28,29^ The activation of type-3 immunity in the gut involves local upregulation of IL-23 and expansion of IL-17-producing type 3 innate lymphoid cells (ILC-3) cells, which circulate and potentially relocate to skeletal sites.^30,31^ Moreover, in a rat model of SpA, IL-23 may activate cells of the innate immunity at the entheses to produce IL-17 and IL-22, both important downstream effector cytokines in the pathogenesis of SpA.^32^ Recent research has demonstrated that normal human entheses also harbour cells of the innate immunity (ILC-3 and Tγ/δ lymphocytes) that are able to produce IL-17 both in an IL-23-dependent and independent manner.^33,34^

It is possible that IBD and SpA share a common initial sequence of events that unfold at the gut mucosa. However, differences in the genetic background, environmental and other as-yet unidentified factors may determine whether the inflammatory process expands and persists locally evolving to clinical IBD and/or relocates and establishes itself at entheseal sites giving rise to clinical SpA.

## DIAGNOSIS

The key to early diagnosis of IBD-SpA is a high level of suspicion of rheumatologists for gastrointestinal symptoms in SpA patients and of gastroenterologists for musculoskeletal symptoms in IBD patients. Several questionnaires^35–37^ and screening tools38 have been proposed to help gastroenterologists identify musculoskeletal involvement in IBD patients and vice-versa, although their performance in clinical practice remains to be validated. Moreover, expert panels including rheumatologists and gastroenterologists have pointed some signs or symptoms that should be regarded as “red flags” suggestive of IBD or SpA and should prompt for further investigation.^11,39–43^ (**Table 1**) On the other hand, simple laboratory studies may be ambiguous and cause diagnostic delays. Indeed, among first-line laboratory tests, only pelvis X-rays carry a high positive predictive value for the diagnosis of sacroiliitis.^44^ In this regard, gastroenterologists requesting an abdominal CT should also look at the sacroiliac joints for structural damage due to sacroiliitis.

**Table 1:** Proposed “red flags” to consider further investigation for concomitant IBD or SpA.

**Red flags for IBD**	**Red flags for SpA**
Chronic diarrhoea for more than 4 weeks	Back pain (for more than 3 months)
Abdominal pain for more than 3 months	Recurrent or chronic (more than 3 months) peripheral joint pain or swelling
Nocturnal diarrhoea or abdominal pain	Inflammatory spinal pain: age at onset younger than 40 years, insidious onset, improvement with exercise, not improvement with rest, pain at night
Rectal bleeding (not due to haemorrhoids)	Finger swelling (ie, dactylitis) ever
Perianal fistula or abscesses, recurrent oral aphthosis	Heel pain (ie, enthesitis) ever
Unexplained constitutional symptoms: weight loss, fever, anaemia	Family history of SpA*
Family history of IBD	

* First- or second-degree relatives with IBD, AS, psoriasis, acute uveitis or reactive arthritis IBD, inflammatory bowel disease; SpA, spondyloarthritis; AS, ankylosing spondylitis.

Other studies, such as C-reactive protein (CRP) and HLA B27 are poorly sensitive or specific.^45–48^ Faecal or serum calprotectin has also been associated with subclinical gut inflammation in SpA patients,^49–52^ although faecal calprotectin levels may be influenced by non-steroidal anti-inflammatory drug use, which is common in SpA patients.^50^ However, in most cases the confirmation of IBD or SpA requires advanced or interventional procedures, such as musculoskeletal MRI, arthrocentesis and synovial fluid examination, gastrointestinal imaging, endoscopy or biopsy. The indication to perform and the interpretation of those tests are determined by the specialist, which highlights the importance of symptom-based referral and the gastroenterologist-rheumatologist collaboration.

Although the ASAS criteria may be helpful in identifying patients with SpA,^7^ their validity has not been assessed in IBD-SpA. In fact, there are some peculiarities in this disease that potentially affect the weight of some items and the sensitivity and specificity of the whole system. These include the lower prevalence of HLA B27 in IBDSpA compared to classic AS,^46–48^ the relative contra-indication of non-steroidal anti-inflammatory drugs (NSAIDs) in patients with IBD^53^ and the lack of specificity of elevated serum CRP, as it may be due to active intestinal inflammation, as well.

## TREATMENT CONSIDERATIONS

Physicians treating patients with IBD-related SpA face two major challenges: although there are plenty of drugs approved separately for the treatment of axial SpA, PsA (a model for peripheral SpA) and IBD, there is almost complete lack of evidence concerning the efficacy of those treatments for the combined disease. Moreover, some of those drugs occasionally have contrasting effects on either disease aspect (**Table 2**).

**Table 2:** Overview of the effects of drugs approved for the treatment of IBD and SpA across the main disease manifestations.

**Drug**	**Crohn’s Disease**	**Ulcerative colitis**	**Axial Disease, Enthesitis, Dactylitis**	**Peripheral Arthritis (PsA)**
**NSAIDs**	**Avoid in active disease**	**Avoid in active disease**	**+**	**+**
**Systemic Glucocorticoids**	**+**	**+**	**-**	**Lowest exposure possible**
**Sulfasalazine**	**+**	**+**	**-**	**+**
**Methotrexate**	**+**	**-**	**-**	**+**
**Leflunomide**	**-**	**-**	**-**	**+**
**Azathioprine**	**+**	**+**	**-**	**-**
**Infliximab Adalimumab**	**+**	**+**	**+**	**+**
**Golimumab**	**-**	**+**	**+**	**+**
**Certolizumab**	**+**	**-**	**+**	**+**
**Etanercept**	**-**	**-**	**+**	**+**
**Ustekinumab**	**+**	**+**	**Enthesitis & dactylitis only**	**+**
**Vedolizumab**	**+**	**+**	**-**	**-**
**Secukinumab Ixekizumab**	**Avoid**	**Avoid**	**+**	**+**
**Tofacitinib**	**-**	**+**	**+**	**+**

NSAIDs are the first-line treatment for axial disease, enthesitis, dactylitis and often for peripheral arthritis according to recent recommendations.^54,55^ However, in a population study their use was associated with a dose-dependent risk for incident IBD.^56^ In IBD patients, NSAIDs have been associated with an increase in disease activity, particularly at high-doses and in patients with CD and colonic involvement.^57,58^ On the other hand, a couple of trials showed that selective cyclooxygenase-2 (COX-2) inhibitors (etoricoxib, celecoxib) in IBD patients was not associated with a greater risk for IBD exacerbation than placebo in the short term.^59,60^ Moreover, in a meta-analysis, the pooled proportion of IBD flare associated with selective COX-2 inhibitors was 14%, with most gastrointestinal symptoms occurring over the first few days to weeks of treatment.^61^ Summarizing, although NSAIDs, particularly selective COX-2 inhibitors, may be used for a short time as a symptomatic treatment in patients with quiescent IBD, their long-term safety is a concern and, thus, they are the least attractive treatment option for the chronic musculoskeletal manifestations of IBD-related SpA.^62^

Glucocorticoids (GC), either topically (oral budesonide) or systemically acting, are an effective therapy for inducing remission in CD,^63,64^ although they are not effective for maintaining remission.^65^ Systemic GC are also appropriate for patients with moderate to severe UC and for those with mild disease not responding to mesalazine. On the other hand, there is little place for GCs in the treatment of SpA. Although a trial of high dose oral GC in axial SpA showed a modest effect,^66^ long-term GC treatment of axial SpA is discouraged.^54,55^ Short-term low-dose oral GCs may be used in case of peripheral arthritis, while local GC injections may be used for the treatment of sacroiliitis, peripheral arthritis or enthesitis.^54,55,67^

Conventional immunomodulatory drugs used either for SpA or IBD include aminosalicylate compounds, methotrexate, leflunomide and azathioprine. Concerning aminosalicylates, both sulfasalazine and mesalazine are effective in UC, but their benefit in CD has not been well documented.^68^ Moreover, as mesalazine is better tolerated than sulfasalazine,^69^ most gastroenterologists would probably favour the former over the latter. This comes in contrast to the rheumatological practice, since only sulfasalazine may have some effect in peripheral arthritis of SpA, for which it is recommended.^54,55,67,70,71^ Conversely, published evidence does not support any benefit of sulfasalazine in reducing pain, disease activity, radiographic progression or improving physical function and spinal mobility in axial SpA.^72,73^

While methotrexate is an established treatment option for CD,^74^ a couple of randomised trials showed no significant benefit over placebo in UC.^75,76^ Concerning axial SpA the evidence supporting its use is very poor^77,78^ and, therefore, it may be considered only for patients who cannot tolerate other drugs with proven efficacy.^54,55^ On the other hand, methotrexate is the first-line treatment for peripheral arthritis of PsA.^67^ Similarly, although leflunomide is efficacious in treating PsA,^79^ it is not efficacious in axial SpA^80^ while limited data for the treatment of IBD show some effectiveness in IBD-related joint pain.^81,82^ Finally, azathioprine, an immunosuppressant beneficial for both CD and UC^83,84^ has no place in the treatment of SpA manifestations.

Considering biologics, there are only few small open label studies assessing their efficacy for the treatment of both aspects of IBD-SpA.^85–87^ In fact, all randomized clinical trials of biologics in axial SpA excluded patients with active IBD.^88–94^ On the other hand, the published evidence on the treatment of peripheral SpA is dominated by trials in PsA, with only a few studies of adalimumab and golimumab in non-psoriatic peripheral SpA. In those studies, though, the proportion of patients with clinical IBD was too low to allow for meaningful conclusions.^95–98^ Conversely, no randomized trial of targeted treatments for CD and/or UC reported rheumatological outcomes.^99–108^ For over a decade TNFα blockade has been the only common second-line targeted treatment for both SpA and IBD. However, direct clinical evidence on the efficacy of TNF inhibitors across both domains of IBD-SpA is very limited. A trial of golimumab and infliximab showed efficacy on both aspects of the disease,^85,86^ while, etanercept is efficacious only for the musculoskeletal, but not gut involvement.^87^ Finally, the various currently available TNF inhibitors differ in terms of approved indications and dosages between SpA and IBD. Indeed, golimumab is not approved for CD, while certolizumab pegol is approved for CD in the US, but not in the EU. Moreover, dosages may differ between rheumatological and gastroenterological indications, with IBD generally requiring higher dosages (**Tables 2 and 3**).

**Table 3:** Dosages of targeted treatments approved for AxSpA/PsA, Crohn’s disease and Ulcerative Colitis.

	**AxSpA & PsA**	**Crohn’s disease**	**Ulcerative Colitis**

**Infliximab**	Loading dosage	Loading dosage			
	5 mg/kg at 0, 2, 6 weeks	5 mg/kg at 0, 2, 6 weeks			

	Maintenance dosage	Maintenance dosage			
	5mg/kg every 6–8 weeks (AxSpA) or 8 weeks (PsA)	5mg/kg every 8 weeks			

**Adalimumab**	40 mg every 2 weeks	Loading dosage 80 mg (week 0), 40mg (week 2) 160 mg (week 0), 80mg (week 2)*	Loading dosage 160 mg (week 0), 80mg (week 2)

		Maintenance dosage 40 mg every 2 weeks** 80mg every 2 weeks or 40mg every week***	Maintenance dosage 40 mg every 2 weeks** 80mg every 2 weeks or 40mg/week***

**Golimumab**	50 mg every month 100 mg every month, if body weight >100kg**		Loading dosage 200 mg (week 0), 100mg (week 2)

			Maintenance dosage 50–100mg† every 4 weeks (EU label) US 100mg every 4 weeks (US label)

**Certolizumab pegol**	Loading dosage	Loading dosage	
	400mg at week 0, 2, 4	400mg at week 0, 2, 4	

	Maintenance dosage	Maintenance dosage	
	200 mg every 2 weeks	200 mg every 2 weeks	

**Ustekinumab**	PsA only: 45mg **sc** at weeks 0, 4, then every12 weeks 90mg **sc** at weeks 0, 4, then every12 weeks, if body weight >100 kg	Loading dosage			
		6mg/kg **iv** (week 0), 90mg **sc** at week 8			

		90 mg every 8–12 weeks **sc** ‡ (EU label) 90 mg every 8 weeks **sc** (US label)			

**Tofacitinib**	5 mg twice daily		Loading dosage
			10 mg twice daily for 8–16 weeks¶

			Maintenance dosage
			5–10 mg twice daily§

* In case fast response is pursued. The only approved loading dosage in the US

** The only approved maintenance dosage in the US

*** In case of inadequate response to the lower dosage (EU label)

† In case of inadequate response to the lower dosage or body weight ≥80kg

‡ In case of inadequate response to the 12-week schedule

¶ In case of inadequate response during the first 8 weeks

§ In case of loss of response dosage may be increased to 10mg twice daily for the shortest time necessary; dosage of the extended-release formulation is not shown

Among biologics with alternative modes of action, the IL-12/23 blocker ustekinumab is effective and has been approved for the treatment of CD, UC and peripheral arthritis, enthesitis and dactylitis of PsA.^105,108–110^ Moreover, a retrospective study of patients with psoriasis or PsA and concomitant IBD treated with ustekinumab for the psoriatic disease showed a benefit for IBD as well.^111^ However, ustekinumab failed to show efficacy in the treatment of axial SpA,^112^ while there are no data on the treatment of non-psoriatic peripheral SpA. Secukinumab is an IL-17 inhibitor which is effective for the treatment of axial SpA, as well as PsA.^93,113,114^ When tried in CD, though, a high rate of adverse events, mainly fungal infections, were reported.^115^ Moreover, there have been several reports of new onset or flaring IBD in patients with SpA, PsA, psoriasis or IBD treated with IL-17 blockers (secukinumab, ixekizumab, brodalumab). A class effect has been suspected and, therefore, IL-17 inhibitors are not currently recommended for patients with known IBD.^116^ However, long-term trial data, as well as meta-analyses showed a low risk of new onset IBD in patients with SpA, PsA or psoriasis treated with IL-17 blockers.^117,118^

The α4β7 integrin blocker vedolizumab is considered gut-specific and is used for the treatment of both CD and UC. A post-hoc analysis of the GEMINI trials of vedolizumab in IBD focusing on joint symptoms showed a lower risk for new-onset joint symptoms in CD, but not in UC.^119^ However, several case series have been published reporting new-onset or flaring musculoskeletal manifestations in IBD patients treated with vedolizumab.^120,121^ In a large cohort of IBD patients treated with vedolizumab, an improvement of joint symptoms was noted in 45% of patients who had such symptoms at baseline. The benefit was greatest in patients with recent-onset articular manifestations and those who attained IBD remission under vedolizumab. On the other hand, 14% of patients, mainly with CD and pre-existing AS, developed new onset joint symptoms under vedolizumab.^122^ These seemingly paradoxical results may actually reflect the potential different mechanisms of arthritis in IBD: a “reactive” mechanism in which suppressing bowel inflammation also benefits the extraintestinal manifestations; and an “autonomous” mechanism, in which once joint inflammation begins, it propagates independently of IBD activity.

Finally, tofacitinib has been approved for UC^123,124^ and PsA,^125,126^ while it has shown efficacy for the treatment of AS.^127^ However, there are almost no data concerning response of joint disease in IBD.^128^ Moreover, compared to PsA, treatment of UC requires higher doses of tofacitinib (**Table 3**) which have been associated with an increased risk of cardiovascular thromboembolic events.^129^

Last but not least, in recent years there is a significant interest in an unexplored field of treatment, the combination therapy. Multidrug therapy seems promising, but concerns for potential adverse events are high. There have been treatment considerations for combining traditional DMARDs with biological DMARDs or two biological DMARDs.^130–134^

Apart from the lack of single drug trials, there is very scarce evidence on how to start or escalate treatments in IBD-SpA. There is only a single investigator-initiated strategy trial involving both gastroenterologists and rheumatologists for the care of patients with IBD-SpA. In this study, treatment decisions were based on the disease state over 3 domains (intestinal, axial or peripheral skeletal) according to which the patient would receive conventional synthetic disease-modifying anti-rheumatic drugs (DMARDs), or adalimumab. Notably, adalimumab was prescribed at the highest dosage, as approved for IBD. The study showed that this approach was associated with a significant improvement after 6 and 12 months in all outcomes measured, including BASDAI, ASDAS-CRP, CDAI, partial Mayo score, BASFI, HAQ, SF-36 and IBDQ.^15^ Although this was a small (N=52) and uncontrolled observational study, it also highlighted the value of collaborative work among physicians with different specialties for the treatment of IBD-SpA.

As evident from the above (**Table 2**) there is only a limited number of advanced treatments that appear safe and effective for all manifestations of IBD-SpA. In real world, though, it is not unusual for patients to have failed them all, making the treatment of IBD-SpA a real challenge for rheumatologists and gastroenterologists. There have been several cases of combining targeted drugs to treat IBD and SpA with often positive results.^130–134^ The main concern, though, is the long-term safety of combining immunomodulatory treatments, which should be addressed in clinical trials and long-term observational studies.

## CONCLUSIONS

Inflammatory bowel disease-related spondyloarthritis is a common and debilitating disease that has not attracted equal scientific and medical attention. It is most important that rheumatologists and gastroenterologists become aware of both aspects of the disease, in order to actively seek for symptoms that will prompt further investigation and early diagnosis. Given the lack of scientific evidence, collaboration between physicians of both specialties is necessary, so that patients receive the appropriate treatments at the optimal dosages for their manifestations and are followed up properly. Regarding research, apart from studying the underlying pathophysiology, it is time that clinical trials include patients with both manifestations, so that the effect of existing and upcoming treatments is evaluated directly and not extrapolated from trials in Rheumatology and Gastroenterology alone. To this end, composite measures of gut and musculoskeletal disease activity should be developed and validated, in order to be used in trials, as well as in every day clinical practice. As this last rheumatological domain is gradually brought to light, it appears that there is still immense room for research and improvement in the care of those patients.
